# TA-MSCs, TA-MSCs-EVs, MIF: their crosstalk in immunosuppressive tumor microenvironment

**DOI:** 10.1186/s12967-022-03528-y

**Published:** 2022-07-16

**Authors:** Zhenghou Zhang, Xiangyu Zhou, Jinshuai Guo, Fusheng Zhang, Yiping Qian, Guang Wang, Meiqi Duan, Yutian Wang, Haiying Zhao, Zhi Yang, Zunpeng Liu, Xiaofeng Jiang

**Affiliations:** 1 Department of General Surgery, The Fourth Affiliated Hospital of China Medical University, Shenyang, Liaoning China; 2 Department of Orthopedics, The Fourth Affiliated Hospital of China Medical University, Shenyang, China

**Keywords:** Immunosuppressive tumor microenvironment, TA-MSCs, Extracellular vesicles, Macrophage migration inhibitory factor, Anti-tumor immune cells, Immunosuppressive cells

## Abstract

As an important component of the immunosuppressive tumor microenvironment (TME), it has been established that mesenchymal stem cells (MSCs) promote the progression of tumor cells. MSCs can directly promote the proliferation, migration, and invasion of tumor cells via cytokines and chemokines, as well as promote tumor progression by regulating the functions of anti-tumor immune and immunosuppressive cells. MSCs-derived extracellular vesicles (MSCs-EVs) contain part of the plasma membrane and signaling factors from MSCs; therefore, they display similar effects on tumors in the immunosuppressive TME. The tumor-promoting role of macrophage migration inhibitory factor (MIF) in the immunosuppressive TME has also been revealed. Interestingly, MIF exerts similar effects to those of MSCs in the immunosuppressive TME. In this review, we summarized the main effects and related mechanisms of tumor-associated MSCs (TA-MSCs), TA-MSCs-EVs, and MIF on tumors, and described their relationships. On this basis, we hypothesized that TA-MSCs-EVs, the MIF axis, and TA-MSCs form a positive feedback loop with tumor cells, influencing the occurrence and development of tumors. The functions of these three factors in the TME may undergo dynamic changes with tumor growth and continuously affect tumor development. This provides a new idea for the targeted treatment of tumors with EVs carrying MIF inhibitors.

## Introduction

The tumor microenvironment (TME) comprises cellular and non-cellular components of the tumoral niche, such as immune inflammatory cells, myofibroblasts, extracellular matrix (ECM) and vascular networks [1]. Formation of TME is a constantly changing complex dynamic process involving remodeling of tumor cells, metabolic changes in cellular components and continuous alterations of ECM [2]. During tumor progression, immune inflammatory cells are stimulated by tumor cells and recruited to the tumor site, thus inducing immune responses that continuously affects tumor growth, leading to the formation of immunosuppressive TME [3]. In most cases, immunosuppressive TME does not inhibit tumor growth, but instead promotes tumor progression. Numerous studies have confirmed that tumor progression is inseparable from the supporting effects of immunosuppressive TME on tumor cell proliferation, survival and invasion [4].

Mesenchymal stem cells (MSCs), a cellular component of TME, are critical for cancer growth and metastasis [5]. Bone marrow is the main established source, but with more comprehensive research, MSCs have been successfully isolated from other tissues and organs, such as adipose tissue, umbilical cord blood, placenta, liver, and pancreas [6]. MSCs are a non-hematopoietic cell type with multiple characteristics, including multi-directional differentiation potential [7], directional migration [8] and immunomodulatory effects [9]. Under chemokine regulation, MSCs home from the blood circulation to TME and are transformed into tumor-associated MSCs (TA-MSCs) following tumor cell education. Although the specific role of MSCs in tumor development remains controversial, TA-MSCs have been extensively shown to promote tumor cell proliferation, migration and survival directly or indirectly by secreting bioactive factors and EVs in TME [10].

Extracellular vesicles (EVs) are particles naturally released by cells that are delimited by lipid bilayers and cannot replicate, including exosomes, microvesicles, membrane vesicles, and apoptotic bodies and other types of membrane vesicles [11]. EVs contain various bioactive substances, predominantly small molecular nucleic acids, proteins and lipids that can regulate the biological functions of recipient cells [12].As one of the cell types with the strong ability to secrete EVs [13], MSCs exert significant effects on other cellular components in TME. MSCs-derived extracellular vesicles (MSCs-EVs) contain part of the membrane and signaling factors from MSCs and therefore display similar cell surface markers and ability to home to TME as their parent cells [14]. MSCs-EVs not only participate in cell–cell communication and signal transduction but also affect gene and protein expression patterns of tumor cells, in turn, affecting the formation of immunosuppressive TME and tumor cells metabolism [15]. Notably, the effects of EVs on tumors are similar to those of MSCs.

Macrophage migration inhibitory factor (MIF), a multifunctional cytokine with unique tautomerase and oxidoreductase activities, is widely expressed in different tissues, organs and TME. Originally, MIF was proposed to be a cytokine released by activated T cells to inhibit random movement of macrophages [16]. MIF is a non-cellular component of TME involved in tumor development [17]. Accumulating research has revealed significant upregulation of MIF in various cancer types, including cervical cancer, breast cancer, prostate cancer, hepatoma, neuroblastoma, colorectal cancer, pancreatic cancer and lymphocytic leukemia [18]. Furthermore, MIF promotes tumor proliferation, migration, invasion, angiogenesis and chemotherapy resistance via binding to different receptors, including CD74, C-X-C motif chemokine receptor 2 (CXCR2), CXCR4, and CXCR7 [19]. In this review, the tumor-promoting effects and related mechanisms of TA-MSCs, TA-MSCs-EVs and MIF as well as their potential interrelationships are comprehensively summarized.

## Tumor-promoting effects and related mechanisms of TA-MSCs, TA-MSCs-EVs and MIF

### Tumor-promoting activity of TA-MSCs, TA-MSCs-EVs and MIF

Analysis of the literature indicates that both MSCs and TA-MSCs can promote tumor progression (Tables 1 and 2).

**Table 1:** The effects of MSCs and MSCs-EVs on tumors

Species	Cell source	Type of tumor	Effects on tumor	experimental type	Reference numbers
Human	BM-MSCs-EVs	PC	Inhibition	In vitro and vivo	[23]
Human	BM-MSCs	MM	Inhibition	In vitro	[41]
Human	AD-MSCs	OC	Inhibition	In vitro	[24]
Human	UC-MSCs	HCC	Inhibition	In vitro	[253]
Human	UC-MSCs	LC	Inhibition	In vitro	[254]
Human	UC-MSCs	BC	Inhibition	In vitro and vivo	[255]
Human	UC-MSCs	Melanoma	Inhibition	In vitro and vivo	[22, 256]
Human	NLT-MSCs	LC	Promotion	In vitro and vivo	[26, 37]
Human	BM-MSCs	MM	Promotion	In vitro and vivo	[91]
Human	BM-MSCs	BC	Promotion	In vitro and vivo	[75, 92, 99, 194]
Human	BM-MSCs	Neuroblastoma	Promotion	In vitro and vivo	[43]
Human	AD-MSCs	BC	Promotion	In vitro and vivo	[76, 77, 88, 257]
Human	hESC-MSCs	BC	Promotion	In vitro	[222]
Human	AD-MSCs	Melanoma	Promotion	In vitro and vivo	[102]
Human	BM-MSCs	CRC	Promotion	In vitro and vivo	[78, 101, 208]
Human	UC-MSCs	LC	Promotion	In vitro	[20]
Human	UC-MSCs	BC	Promotion	In vitro and vivo	[223]
Human	UC-MSCs	GC	Promotion	In vitro and vivo	[21]
Human	BM-MSCs	CML	Promotion	In vitro and vivo	[86]
Human	BM-MSCs	PCa	Promotion	In vitro and vivo	[100, 113]
Human	BM-MSCs	GC	Promotion	In vitro and vivo	[196, 199, 208, 218]
Human	BM-MSCs	OS	Promotion	In vitro and vivo	[225, 231]
Human	BM-MSCs	Glioma	Promotion	In vitro	[227]
Human	BM-MSCs	Sacoma	Promotion	In vitro and vivo	[230]
Human	Oral-MSCs	KS	Promotion	In vitro and vivo	[232]
Human	AD-MSCs-EVs	BC	Promotion	In vitro	[44]
Human	BM-MSCs-EVs	NPC	Promotion	In vitro and vivo	[50]
Human	BM-MSCs-EVs	BC	Promotion	In vitro and vivo	[52, 92]
Human	BM-MSCs-EVs	CLL	Promotion	In vitro	[67]
Human	BM-MSCs-EVs	LC	Promotion	In vitro and vivo	[81]
Human	BM-MSCs-EVs	CRC	Promotion	In vitro and vivo	[101]
Human	UC-MSCs-EVs	GC	Promotion	In vitro and vivo	[80, 89]
Human	UC-MSCs-EVs	LC	Promotion	In vitro	[20]
Mouse	BM-MSCs	LC	Promotion	In vitro and vivo	[114, 158, 206]
Mouse	BM-MSCs	MM	Promotion	In vivo	[175]
Mouse	BM-MSCs	Glioma	Promotion	In vitro and vivo	[224]
Rabbit	BM-MSCs	PCa	Promotion	In vitro and vivo	[197]
Rat	BM-MSCs	Glioma	Promotion	In vitro and vivo	[226]

**Table 2:** The effects of TA-MSCs and TA-MSCs-EVs on tumors

Species	Cell source	Type of tumor	Effects on tumor	experimental type	Reference numbers
Human	GA-MSCs	Glioma	Promotion	In vitro and vivo	[48, 98]
Human	UC-MSCs	Liver cancer	Promotion	In vitro	[25]
Human	LC-MSCs	LC	Promotion	In vitro and vivo	[26, 37]
Human	BM-MSCs	PCa	Promotion	In vitro	[27]
Human	PCa-MSCs	PCa	Promotion	In vitro	[113]
Human	CRC-MSCs	CRC	Promotion	In vitro and vivo	[28, 65]
Human	BC-MSCs	BC	Promotion	In vitro	[30]
Human	CeCa-MSCs	CeCa	Promotion	In vitro	[36]
Human	GC-MSCs	GC	Promotion	In vitro and vivo	[39, 49, 79, 151, 167, 183, 204, 217, 218, 258]
Human	AML-MSCs	AML	Promotion	In vitro and vivo	[115, 165, 259, 260]
Human	CML-MSCs	CML	Promotion	In vitro	[40, 47]
Human	ALL-MSCs	ALL	Promotion	In vitro and vivo	[201]
Human	MM-MSCs	MM	Promotion	In vitro and vivo	[41, 66, 90, 91, 131]
Human	NB-MSCs	NB	Promotion	In vitro and vivo	[43]
Human	OC-MSCs	OC	Promotion	In vitro and vivo	[87, 96, 97, 174]
Human	BC-MSCs-EVs	BC	Promotion	In vitro and vivo	[122]
Human	MM-MSCs-EVs	MM	Promotion	In vitro and vivo	[90]
Human	ATRT-MSCs-EVs	ATRT	Promotion	In vitro and vivo	[45]
Human	OSCC-MSCs-EVs	OSCC	Promotion	In vitro	[46]
Human	CLL-MSCs-EVs	CLL	Promotion	In vitro	[67]
Mouse	BC-MSCs	BC	Promotion	In vitro and vivo	[184]
Mouse	MM-MSCs-EVs	MM	Promotion	In vitro and vivo	[91]

It can be found from the table that the effects of MSCs from different normal tissues on tumors are different. For example, some studies have found that UC-MSCs can promote the progression of gastric cancer and lung cancer [20, 21], but some studies have reported that UC-MSCs-EVs can inhibit the metastasis of melanoma [22]. Similar differential results were reported in both BM-MSCs [23] and AD-MSCs [24]. The reasons for these different results may include individual differences in cell host sources and different experimental conditions in different research groups.

MSCs are educated to evolve into a tumor-promoting TA-MSC phenotype in TME. This process triggers alterations in the protein and gene expression profiles of MSCs, in turn, promoting tumor cell proliferation and invasion [25]. For instance, MSCs derived from normal lung tissue stimulated by TME are transformed into TA-MSCs expressing high levels of gremlin 1 (GREM1), lysyl oxidase-like 2 (LOXL2), integrin α 11 (ITGA11), which promote invasion and metastasis of lung cancer cells via stromal pro-metastatic signaling [26]. Similarly, prostate cancer cells educate MSCs to evolve into non-carcinoma-associated fibroblasts (CAFs) phenotype TA-MSCs with increased Fibroblast growth factor 2 (FGF2), osteopontin (OPN) and interleukin-8 (IL-8) levels and reduced secretion of soluble fms-like tyrosine kinase-1 (sFlt-1), promoting tumor cells migration [27]. TA-MSCs-derived extracellular vesicles (TA-MSCs-EVs) could also promote tumor growth and metastasis by regulating the functions of cellular and non-cellular components and directly interacting with tumor cells, augmenting epithelial-mesenchymal transformation (EMT) [28] and angiogenesis [29] with concomitant inhibition of tumor apoptosis [30]. Extensive evidence supports a stimulatory role of MIF in tumor progression although a few studies have described an inhibitory effect [31]. After binding to target cell surface receptors, MIF could directly promote tumor cell proliferation, migration and invasion by activating downstream signaling pathways [32, 33]. In addition to direct effects, MIF is reported to facilitate tumor progression by enhancing angiogenesis and chemotherapy resistance [34, 35].

### Tumor-promoting mechanisms of TA-MSCs, TA-MSCs-EVs (Fig. 1) and MIF (Fig. 2)

**Fig. 1:**
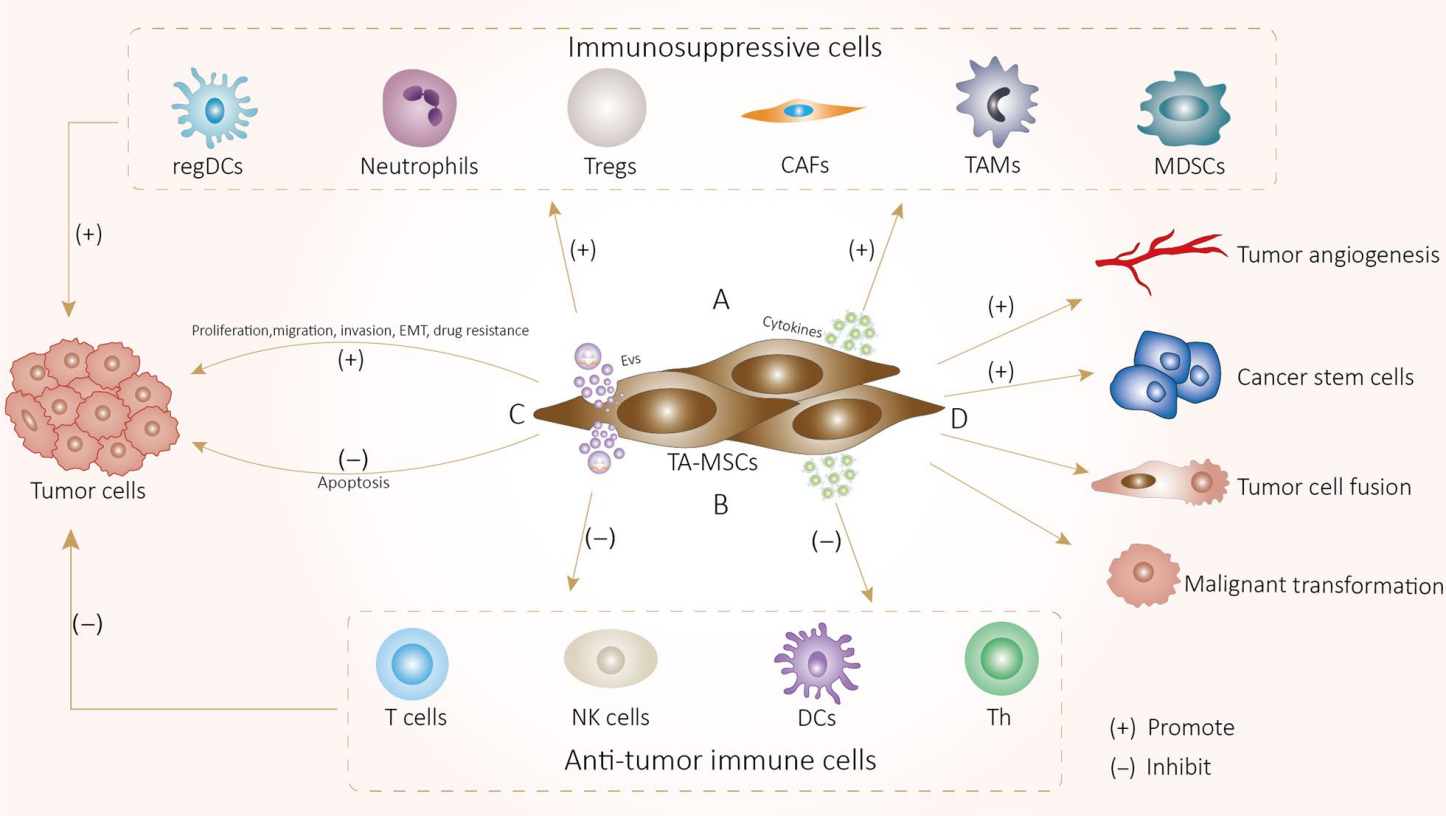
A TA-MSCs promote immunosuppressive cells by secreting EVs and cytokines to indirectly promote tumor progression. B TA-MSCs inhibit anti-tumor immune cells by secreting EVs and cytokines to indirectly promote tumor progression. C TA-MSCs directly promote tumor progression by promoting tumor cells proliferation, migration, invasion. EMT and drug resistance, and inhibiting apoptosis. D TA-MSCs indirectly promote tumor progression by promoting tumor stem cells formation, tumor angiogenesis, fusion with tumor cells and spontaneous malignant transformation

**Fig. 2:**
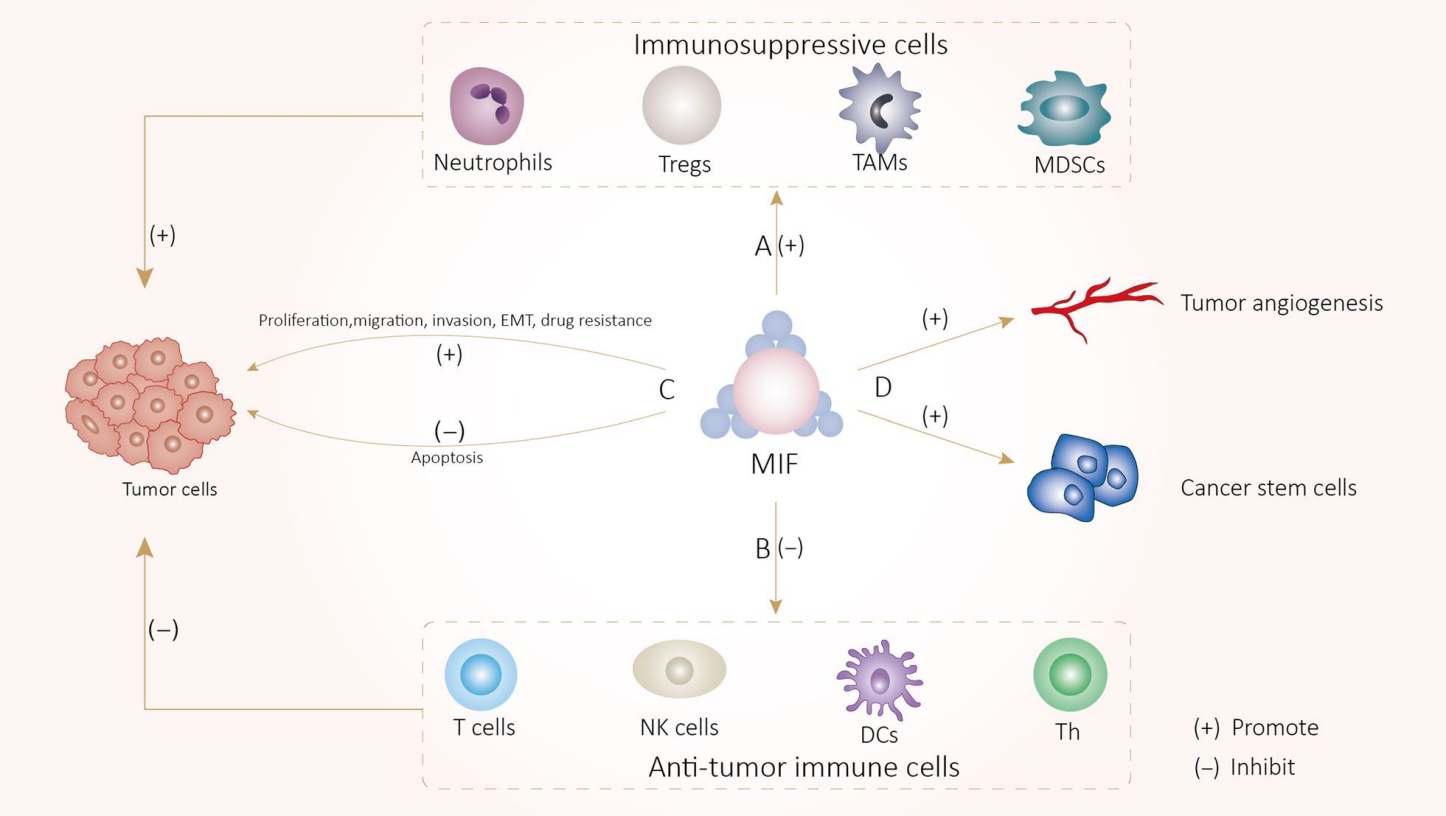
A MIF indirectly promotes tumor progression by promoting immunosuppressive cells. B MIF indirectly promotes tumor progression by inhibiting anti-tumor immune cells. C MIF directly promote tumor progression by promoting tumor cells proliferation, migration, invasion, EMT and drug resistance, and inhibiting apoptosis

TA-MSCs and TA-MSCs-EVs suppress the anti-tumor immune response by not only inhibiting the functions of T cells [36], natural killer cells (NK) [37] and dendritic cells (DCs) [38] but also increasing the number and function of immunosuppressive cells, such as tumor-associated macrophages (TAMs) [39], CAFs [29], and myeloid-derived suppressor cells (MDSCs) [40], leading to tumor immune escape. In addition, TA-MSCs influence the formation of immunosuppressive TME by secreting tumorigenic cytokines and inflammatory factors [41]. MIF can also promote tumor progression by similar mechanism, the mechanisms by which TA-MSCs, TA-MSCs-EVs and MIF promote tumor development are described below.

#### Direct effects of TA-MSCs, TA-MSCs-EVs and MIF on tumor

##### Enhancement of proliferation, migration, and invasion of tumor cells

MSCs from multiple myeloma (MM) (MM-MSCs) patients are reported to activate mitogen-activated protein kinase (MAPK) (extracellular signal-regulated kinase (ERK1/2) and c-jun N-terminal kinase (JNK)) signal transduction, which stimulates translation initiation (TI) factors (eukaryotic translation initiation factor 4E (eIF4E) and eIF4GI) and subsequent upregulation of eIF4E/eIF4GI-dependent protein targets, including hypoxia-inducible factor 1α (HIF1α), mothers against decapentaplegic homolog 5 (Smad5), nuclear factor-κB (NF-κB), c-Myc and cyclin D1, promoting proliferation, migration, invasion, survival and chemotherapy resistance of MM cells [41]. A similar mechanism has been confirmed for MSCs derived from lung cancer (LC-MSCs) [42]. Other studies report that TA-MSCs secrete various cytokines, such as platelet-activating factor (PAF), asporin (ASPN), and IL-6, which enhance proliferation, migration and invasion properties of tumor cells via activation of multiple critical downstream pathways, including signal transducer and activator of transcription 3 (STAT3), protein kinase B (PKB/AKT), janus kinase 2 (JAK2) and ERK1/2 [29, 42, 43].

In terms of the effects of TA-MSCs-EVs, breast cancer-derived MSCs-EVs (BC-MSCs-EVs) activate the WNT signaling pathway in vitro, leading to cancer cell migration [44]. Furthermore, TA-MSCs transport microRNAs (miR) into tumor cells, such as miR-155, miR-8485, miR-221, miR-1587, which enhance proliferation, migration and invasion directly or indirectly by targeting downstream genes or proteins associated with tumor growth and metastasis (SMARCA4, BCR-ABL1 and transforming growth factor β1(TGF-β1)) [45–49]. In addition, TA-MSCs-EVs have the ability to transport other small molecule bioactive compounds. MSCs-EVs molecules have been shown to transfer FGF19 to nasopharyngeal cancer cells, in turn, activating a FGF19-FGFR4-dependent ERK signaling pathway that stimulates cancer cell proliferation and migration [50]. Similarly, chronic myelomonocytic leukemia-derived MSCs-EVs (CML-MSCs-EVs) transfer tissue factor (TF) to hematopoietic stem cells (HSCs) to facilitate tumor progression [51]. Proteomic analysis has revealed more than 150 different proteins in EVs secreted by human serum-derived MSCs (SD-hMSCs), the majority of which support tumor growth, including platelet-derived growth factor receptor β (PDGFRβ), tissue inhibitor of metalloproteinase-1 (TIMP-1) and TIMP-2 [52].

Similar with TA-MSCs and TA-MSCs-EVs, MIF can also activate multiple signaling pathways via binding to different receptors on the tumor cell surface and affects the expression of a range of downstream proteins and genes, in turn, influencing the proliferation, migration and invasion of tumors.

High expression of MIF in tumors activates the PI3K/AKT pathway via interactions with CD74 and CXCR7 [53], thereafter inhibiting NR3C2 [32], p53 [33] and p27 [54] expression and upregulating downstream targets, including cyclin D1, cyclin-dependent kinase (CDK), MMP-7, c-Myc, c-Jun, MMP-9 and VEGF [55–57], thus promoting tumor growth, metastasis and survival while inhibiting apoptosis.

MIF activates the MEK/ERK pathway [58] to promote tumor cell proliferation, survival and metastasis in a dose-dependent manner by downregulating p53 [33] and upregulating BCL2 and CD74 [57].

MIF has additionally been shown to activate the signal transduction of lysophosphatidic acid (LPA) associated with the Rho-dependent pathway, resulting in focal adhesion kinase (FAK) phosphorylation and LPA-induced integrin B1 and MMP-13 expression, which drive invasion and metastasis of colon cancer [59]. Similarly, MIF promotes cervical cancer (CeCa) cell proliferation through increasing cyclin D, p16 and c-Myc and decreasing cyclin E expression in tumor cells and upregulates Src and FAK expression to promote tumor cell migration [60].

MIF also induces NF-κB activation [55, 61] through interactions with CD74 and enhances NF-κB target gene expression, including cellular inhibitors of apoptosis 2 (c-IAP2), Bcl-xl, MMP-2, uridylyl phosphate adenosine (uPA) and HIF-1α, facilitating the proliferation and migration of tumor cells [62, 63].

Importantly, MIF overexpressed in brain tumor-initiating cells (BTIC) could transform benign glial cells into BTIC through interactions with p53 and further promote BITC proliferation by direct inhibition of p53, resulting in initiation and progression of malignant brain tumors [64].

The collective studies clearly demonstrate that TA-MSCs, TA-MSCs-EVs and MIF drive proliferation, migration and invasion of tumor cells through direct information exchange between cells.

##### Inhibition of tumor cell apoptosis

TA-MSCs protect tumor cells from spontaneous and chemotherapy-induced apoptosis, thus enhancing survival [43]. Recent research suggests that colorectal cancer (CRC)-MSCs inhibit CRC cell senescence via the p53-p21 pathway, leading to stimulation of CRC development in vivo [65]. MM-MSCs protect MM cells from chemotherapy-induced apoptosis via activation of NF-κB and its downstream pathways [66].In addition, MSCs-EVs isolated from chronic lymphocytic leukemia (CLL) patients (CLL-MSCs-EVs) could additionally protect CLL B cells from spontaneous and drug-induced apoptosis in a dose-dependent manner, enhancing their survival and tumor progression through pathways related to activation of B-cells receptors [67].

In an earlier study, MIF gene knockout was shown to enhance the expression of apoptosis-related proteins, including Bcl-2, p-caspase-3 and Bax, in hepatoma cells [68]. Consistently, MIF expression is significantly correlated with susceptibility of colon cancer cells to hypoxia-induced apoptosis [69]. Follow-up studies indicate that lung injury induces an increase in MIF expression, which protects lung cancer cells from apoptosis and promotes tumor proliferation [70]. Lue et al*.* [71] demonstrated that MIF protects tumor cells from apoptosis, promoting survival through activating the PI3K/AKT pathway mediated by CD74. More recent experiments provide evidence that MIF activates NF-κB through binding to CD74 to regulate mitochondrial dynamics and stability, thus facilitating carcinogenesis via suppression of apoptosis [72]. Further studies have additionally confirmed that MIF inhibits p53-mediated apoptosis. For example, MIF downregulates p53 in ultraviolet B (UVB)-induced non-melanoma skin cancer (NMSC) cells, leading to stimulation of tumor cell proliferation [73]. Chronic UVB exposure induces increased production of MIF, which, in turn, inhibits apoptosis by suppressing p53 and its downstream genes, Bax and p21, resulting in photocarcinogenesis [74]. It is not difficult to find that TA-MSCs, TA-MSCs-EVs and MIF show a certain degree of similarity in the mechanism of inhibiting tumor cell apoptosis, which is reflected in the coincidence of signaling pathway, tumor type and apoptosis-related proteins.

##### Promotion of tumor EMT

EMT refers to the biological process by which epithelial cells are transformed into the mesenchymal phenotype. EMT is an important biological process for tumor cells derived from epithelial cells to acquire migration and invasion ability. Early studies have shown that MSCs induce EMT in BC cells and destroy intercell adhesion via activation of a disintegrin and metalloproteinase 10 (ADAM10), promoting cancer cell migration [75]. Similarly, breast cancer derived MSCs (BC-MSCs) promote EMT and maintain a stable mesenchymal state of BC cells via paracrine mechanisms or induce BC cells to autocrine TGF-β1, zinc finger E-box-binding protein 1(ZEB1)/miR-200 regulatory loop, TGF-β-Smad3 signaling pathway may be involved in this process [76, 77]. TNF-α induces secretion of high levels of C–C chemokine ligand 5 (CCL5) by MSCs, which interacts with its receptor C–C chemokine receptor type 1 (CCR1) expressed in colon cancer cells. EMT, proliferation and invasion of cancer cells are subsequently enhanced through upregulation of the CCL5/CCR1/β-catenin/slug pathway [78]. Furthermore, gastric cancer (GC)-MSCs-derived IL-15 is reported to promote EMT of GC cells via STAT3 activation [79].

MSCs-EVs induce EMT of GC and lung cancer cells in a manner dependent on activation of TGF-β1 and Akt pathways [20, 80] and that of non-small cell lung cancer (NSCLC) cells through miR-21-5p delivery [81].

Similar mechanisms have also been found in MIF and tumor-related studies. High expression of MIF in salivary adenoid cystic carcinoma (SACC) cells promotes migration and invasion via increasing EMT [82]. Similarly, overexpressed MIF in PC cells enhances ZEB1/2 and reduces miR-200b expression, in turn, inducing EMT, tumor growth and metastasis in vivo [35]. In NSCLC, ionizing radiation (IR) induces dissociation of the MIF-ribosomal protein S3 (rpS3) complex and enhances MIF expression, which activates the NF-kB signal and triggers EMT of tumor cells [83]. MIF suppresses NR3C2 expression through upregulating miR-301b, thereby promoting EMT in pancreatic cells. Furthermore, this regulatory process potentially involves the PI3K/AKT pathway [32]. High expression of MIF and CXCR4 in glioblastoma cells under hypoxia conditions is reported to promote EMT of glioblastoma cells via a MIF-CXCR4-AKT pathway [84]. Interestingly, MIF also induces malignant MET of normal fibroblasts via upregulation of the TERT gene, leasing to enhanced tumorigenesis [85].

##### Induction of chemotherapeutic resistance

MSCs mediate protection of chronic myelocytic leukemia (CML) cells from imatinib-induced apoptosis through reducing the activity of caspase-3 and expression of anti-apoptotic protein Bcl-xl via a CXCL12/CXCR4 axis [86]. Similar results have been reported for TA-MSCs. For instance, oral carcinoma-MSCs induce IL-6/protein-rich tyrosine kinase-2 (PYK2)-dependent chemotherapy resistance via secretion of CCL2/CCL5 [87]. MM-MSCs activate NF-κB signaling in MM cells in an autophagy-dependent manner, which induces resistance to cell cycle arrest and apoptosis caused by melphalan or doxorubicin [66]. STAT3 and ERK1/2 signaling are additionally involved in TA-MSCs induced chemotherapy resistance [43]. Furthermore, direct contact with MSCs leads to activation of tyrosine kinase Src, loss of phosphatase and tensin homolog (PTEN) and stimulation of the phosphatidylinositol 3-kinase/protein kinase B (PI3K/AKT) pathway in BC cells, which induces resistance to trastuzumab [88].

Interestingly, a study by Crompot et al. [67] reported that CLL-MSCs-EVs drive resistance of CLL B cells to the common chemotherapy drugs fludarabine, ibrutinib, idelalisib and venetoclax, etc.) [67]. GC-MSCs-EVs up-regulated the expression of multi-drug resistance- associated proteins, such as multidrug resistance protein (MRP) and lung resistance protein (LRP), in GC cells by activating the kinase cascade of calcium/calmodulin-dependent protein kinases (CaM-K) and Raf/MEK/ERK, inhibit apoptosis caused by 5-fluorouracil (5-FU), and induce resistance to 5-FU [89]. MM-MSCs-EVs molecules transport PSMA3 and PSMA3-AS1 into tumor cells. Consequently, resistance to proteasome inhibitors (PI) mediated by the PSMA3-AS1/PSMA3 signaling pathway is induced, resulting in lower progression-free and overall survival [90]. Survival-related signaling pathways, such as JNK, p38, p53, and Akt, are additionally involved in MM-MSCs-EVs-induced bortezomib resistance [91]. Similarly, BC-MSCs-EVs containing miR-222/223 are efficiently internalized by tumor cells, leading to cancer cell dormancy and chemotherapy resistance and consequently, poor prognosis and recurrence [92].

A close relationship between MIF and tumor drug resistance has been established. MIF-deficient MM cells are reported to develop sensitivity to chemotherapy upon co-culture with BM-MSCs in vivo and in vitro. MIF inhibitors additionally increase the sensitivity of MM cells to chemotherapy [85]. Overexpression of MIF enhances the resistance of PC cells to gemcitabine through promoting ZEB1/2 and reducing miR-200b expression, which contributes to poor prognosis [35]. Subsequent experiments suggest that MIF suppresses the sensitivity of PC cells to gemcitabine through a MIF-miR-301b-NR3C2 pathway [32]. Furthermore, MIF upregulates anti-apoptotic proteins (Bcl-xl and Bcl-2) and downregulates pro-apoptotic proteins, such as Bax and Bad, through the RAS/MAPK pathway, promoting chemotherapeutic resistance of BC cells in vivo and in vitro [93]. High expression of MIF in KRAS mutant CRC cells activates STAT3 and MAPK, which upregulate cyclin D1 expression and reduce caspase 3 activity, resulting in resistance of CRC cells to the MEK blocker refametinib [94].

In terms of promoting tumor drug resistance, TA-MSCs, TA-MSCs-EVs and MIF can promote tumor drug resistance by activating the same signaling pathway and regulating similar related protein expression, such as STAT3, MAPK, Bcl etc.

##### Promotion of the formation and function of cancer stem cells (CSCs)

MSCs enhance the formation and function of CSCs, which also play an important role in tumor progression [95]. Several cytokines and functional proteins expressed by TA-MSCs, such as IL-6, bone morphogenetic protein 2 (BMP2) and BMP4, drive CSCs formation [42, 96]. Coffman et al*.* [97] demonstrated that BMP4 derived from OC-MSCs upregulates Hedgehog (HH) expression in ovarian cancer cells. IL-6 secreted by MSCs promotes the expression of CD133 and activation of JAK2 signal transducer and activator of STAT3 pathway in CRC cells, thus enhancing cancer progression by increasing the number of CSCs [28]. Similar results have been reported from studies on glioma and BC whereby TA-MSCs-secreted IL-6 increased the number of CSCs in tumor tissue and promoted proliferation and self-renewal of tumor cells through activating the STAT3 signal pathway [98, 99]. Prostate cancer-derived MSCs (PCa-MSCs) downregulate androgen receptor (AR) signaling in prostate cancer cells by expressing high levels of CCL5, which induce an increase in the prostate CSCs population, resulting in upregulation of matrix metalloproteinase-9 (MMP-9), ZeB-1, CD133 and CXCR4 and enhanced metastatic ability [100]. Li and co-workers showed that MSCs-EVs promote colon cancer stem cell-like traits and increase the population of CSCs via transfer of miR-142-3p [101]. MSCs-EVs also facilitate acquisition of a CSCs-like phenotype on MM cells and enhance selective enrichment properties of CSCs [102].

Several studies have shown that MIF affects stem cell differentiation and proliferation. For example, MIF is reported to promote neural stem cell proliferation [103] and neuronal differentiation through activation of the Wnt/β-catenin pathway [104]. Recent experiments suggest that MIF is significantly correlated with the proportion of CSCs in tumor tissues [105]. CSCs-derived MIF enhances the immunosuppressive function of MDSCs in a CXCR2-dependent manner, thereby promoting the proliferation, survival and immune evasion of glioblastoma cells [106]. Furthermore, MIF promotes CSCs differentiation, which enhances the chemoresistance of MM cells [107]. Bitarte et al*.* [108] reported that miR-451 downregulation directly targets MIF involved in COX-2 expression that promotes Wnt activation to drive CSCs growth.

In conclusion, TA-MSCs TA-MSCs-EVs and MIF effectively promote the occurrence and development of cancer through interactions with CSCs.

#### TA-MSCs, TA-MSCs-EVs and MIF regulate the immunosuppressive properties of TME

TA-MSCs and TA-MSCs-EVs have stronger immunosuppressive effects than normal MSCs, including inhibition of anti-tumor immune cells and promotion of immunomodulatory cells, which effectively contribute to malignant tumor progression. Notably, MIF can also induce immunosuppression by regulating various cell types in TME, including T cells, NK cells, DCs, MDSCs, TAMs, and Tregs, triggering immune escape by not only preventing tumor cells from being killed by anti-tumor immune cells but also promoting formation of immunosuppressive cells or enhancing their function, and ultimately, tumor progression.

##### TA-MSCs, TA-MSCs-EVs and MIF inhibit anti-tumor immune cells

T cells. T cells, an important component of TME and the immune system, mainly exert anti-tumor effects. Extensive studies suggest that MSCs inhibit both T cells proliferation [109] by secreting Prostaglandin E2 (PGE2) and programmed death ligand-1 (PD-L1) [110] and T cells anti-tumor immunity [38] by secreting immunosuppressive cytokines, such as IL-4 and IL-10 [111], which are not necessarily related to the MSC source. TA-MSCs also promote the formation and maintenance of immunosuppressive TME via inhibition of anti-tumor T cells. For example, NB-MSCs have been shown to significantly suppress proliferation of activated T cells [112]. Pancreatic cancer (PC)-MSCs and melanoma-MSCs exert similar effects on T cells [38, 113]. In a previous study, LC-MSCs reduced the number of CD4^+^ T cells producing IL-17 and cytotoxic CD8^+^ T lymphocytes (CTLs) and suppressed the expression of cytotoxic molecules (Fas ligand (FasL), perforin (PFP) and CD107) in CTLs that mediate anti-tumor immune responses [114]. Recent research has disclosed that acute myeloid leukemia (AML)-MSCs are able to block the production of leukemia-reactive CTLs via a novel COX2/PG/NR4A/WNT signaling pathway, resulting in attenuation of anti-tumor immunity [115]. CD39 and CD73 ectonucleotidases on the cervical cancer-derived MSC (CeCa-MSC) surface efficiently hydrolyze ATP, ADP and AMP nucleotides to generate adenosine (Ado), which inhibits the proliferation and activation of CTLs as well as anti-tumor immune responses [36].

Previous experiments clearly demonstrate that MSCs-EVs molecules inhibit differentiation and activation of T cells in addition to reducing T cells proliferation and interferon-γ (IFN-γ) release [116]. Recently, MSCs-EVs were shown to significantly suppress CD4^+^ T cells proliferation, IL-17 and IFN-γ levels and enhance TGF-β and IL-10 secreted by T cells [117], which could be attributed to miR-223 delivery [118]. MSCs-EVs also inhibit T cells proliferation and infiltration [119] via TGF-β and adenosine signaling [120] concomitant with a decrease in the percentage of CD4^+^ and CD8^+^ T cells subsets [121]. Furthermore, both BC-MSCs and BC-MSCs-EVs could inhibit production of IFN-γ by CD4^+^ and CD8^+^ T cells, thus thwarting the anti-tumor immune response [122].

MIF downregulates NKG2D to inhibit the antitumor immunity of CD8^+^ T cells [123], inducing immune escape of glioma cells [124]. Furthermore, high levels of MIFs protect BC cells from immunogenic cell death (ICD) and inhibit the antitumor immune response mediated by T cells producing IFN-γ [125]. Similarly, neuroblastoma cells secreting MIF inhibit T cell proliferation and induce cell death through an IFN-γ pathway, which eliminates activated T cells from TME, thus contributing to tumor cell escape from immune surveillance [126]. In an earlier study by Zhou and co-workers, production of high amounts of MIF by neuroblastomas (NB) led to a decrease in the number and infiltration of CD8^+^ and CD4^+^ T cells, and ultimately, suppression of antitumor immunity [127]. MIF has additionally been shown to inhibit the activation of CD4^+^, CD8^+^ T cells and CTLs in the tumor regions of cancer-bearing mice [128]. Downregulation of MIF in BC is associated with a significant increase in infiltration and anti-tumor functions of CD4^+^ and CD8^+^ T cells [129].

Above studies have shown that TA-MSCs, TA-MSCs-EVs and MIF enhance the immunosuppressive activity of tumor microenvironmentnot by inhibiting the number and function of CD4^+^ and CD8^+^ T cells and reducing the expression of IFN-γ in T cells, which can significantly inhibit the anti-tumor immune responses of T cells.

Nature killer (NK) cells. NK cells are major tumor suppressor cells. The CD56^bright^ subsets play an immunomodulatory role through secretion of cytokines and CD56^dim^ subsets exert cytotoxic effects through cell degranulation [130]. Recent studies have reported negative effects of MSCs on NK cells proliferation. Conditioned medium (CM) of MSCs has been shown to induce a significant reduction in the number of NK cells in metastatic tissues of lung cancer, leading to inhibition of anti-tumor cytotoxicity [114]. TA-MSCs also inhibit NK cells-mediated anti-tumor immunity through simultaneously suppressing immunomodulatory factor secretion and degranulation of NK cells [131]. LC-MSCs induce a decrease in NK cells number while significantly attenuating anti-tumor cytotoxicity in an NO- and indoleamine 2,3-dioxygenase (IDO)-dependent manner [114]. Furthermore, by secreting IL-6 and PGE2, squamous cell lung carcinoma-derived MSCs (SCC-MSCs) not only regulate the immunomodulatory effects of CD56^bright^ NK cells through inhibition of IFN-γ and TNF-α secretion but also downregulate NK cells-activating receptors and block CD56^dim^-mediated NK cells degranulation to inhibit the cytotoxicity of NK cells [37]. MSCs-EVs molecules additionally inhibit proliferation, activation, and cytotoxicity of NK cells through activation of downstream TGF-β/Smad2/3 signaling mediated by latency-associated peptide (LAP), thrombospondin 1 (TSP1) and TGF-β delivery [132].

Besides, MIF inhibits antitumor immunity through downregulating NKG2D on NK cells at the transcriptional level, in turn, impairing NK cells cytotoxicity towards ovarian cancer cells [123, 124]. Melanoma-derived MIF suppresses NK cells-mediated killing of uveal melanoma cells, thus maintaining an immunosuppressive TME [133]. A significant negative correlation between serum MIF and NK cells levels has recently been reported in ovarian cancer patients before and after chemotherapy, suggesting that MIF inhibits the production and activation of NK cells [134]. On the other hand, Loyon et al*.* shown that IL-21-induced NK cells affect CD4^+^T cell priming by secreting MIF [135]. Additionally, hypoxia is reported to promote secretion of MIF in NK cells and induce apoptosis of leukemia target cells [136]. Current researchs show that TA-MSCs, TA-MSCs-EVs and MIF inhibit NK cells cytotoxicity and degranulation, but there are few studies on the specific mechanisms, which need to be further elucidated.

Dendritic cells (DCs). DCs are the most powerful antigen-presenting cells that activate anti-tumor immunity through stimulating naive T cells and specific T cells proliferation to inhibit tumor progression during the early stages of tumorigenesis. LC-MSCs significantly reduce the number of DCs by producing tumor necrosis factor (TNF)-α [114]. Melanoma-MSCs suppress the expression of cystathionase in DCs through the IL-10-STAT3 pathway, thus blocking export of cysteine from DCs to T cells, leading to reduced proliferation and effector function of T cells [38, 137]. MSCs also inhibit maturation and typical functions of DCs (such as IL-12 production and the ability to prime T cells) through release of PGE2 [138] and TNFα-stimulating gene (TSG)-6 [139]. Regulatory DCs (regDCs), a distinct DCs population differentiated from mature DCs (mDCs), is characterized by limited T cells proliferation, high endocytotic capacity, low immunogenicity, and strong immunoregulatory function [140]. MSCs induce differentiation of mDCs into regDCs via paracrine hepatocyte growth factor (HGF) [141]. Additionally, regDCs are generated by MSCs from hemopoietic progenitor cells (HPCs) synergistically via the Notch and TGF-β signaling pathways [142]. Zhao et al. [143] showed that CML-MSCs induce differentiation of mDCs into regDCs, which inhibit proliferation of T cells through both TGF-β1 and production of Tregs or T cells anergy. Shahir and co-workers [144] reported that MSCs-EVs inhibit DCs maturation and IL-6 release while increasing IL-10 and TGF-β release.

A number of researchers suggest that MIF secreted by glioblastoma inhibits migration and maturation of DCs, suppressing the anti-tumor immune response [145]. MIF not only suppresses DCs maturation and activation but also significantly impairs their ability to activate cytotoxic T cells killing function, facilitating metastatic melanoma progression [146]. Furthermore, MIF has been shown to inhibit migration of both immature DCs (iDCs) and mDCs, meanwhile impair the expression of co-stimulatory markers [145]. In a mouse model of BC, MIF depletion led to an increase in the abundance and activation of DCs, further confirming that MIF mediates tumor growth promotion through DCs inhibition [125]. Interestingly, inhibition of MIF resulted in functional reversion of MDSCs from an immunosuppressive to immunostimulatory DCs-like phenotype [147].

TA-MSCs, TA-MSCs-EVs and MIF show similar inhibitory effects on DCs, they can inhibit DCs recruitment into immunosuppressive TME, meanwhile suppress maturation and activation of DCs, thus indirectly promoting tumor progression.

T helper cells (Th). Th is one of the T cells subsets that includes Th1, Th2 and Th17 (IL-17-producing effector T cells). Th1 and Th2 are often in a state of balance and disruption of this equilibrium is proposed to affect tumor progression. Th2 mainly plays a promote-tumor role while Th1 and Th17 exert anti-tumor effects [148, 149]. In an earlier study, in vivo injection of multipotent MSCs derived from human-induced pluripotent stem cells (huiPS-MSCs) significantly reduced Th1 in mouse spleen and peripheral blood mononuclear cells (PBMCs) [150]. Similarly, GC-MSCs show the ability to not only reduce the Th17 level in PBMCs but also inhibit Th17 proliferation [151].

MSCs-EVs also promote transformation of Th1 into Th2 and reduce the ability of T cells to differentiate into Th17 [152]. Another recent study has reported that MSCs-EVs inhibit the differentiation of tumor-suppressing Th1 and Th17 [117].

Multiple studies have demonstrated that the MIF level in TME is positively correlated with Th2 and negatively correlated with Th1, although the specific mechanisms are yet to be clarified [153–155]. A high level of MIF in TME is proposed to induce a change in the balance of Th1/Th2 differentiation, leading from Th to Th2 phenotypic differentiation [154]. In addition, MIF significantly enhances lymphocyte production of Th2 cytokines, such as IL-2, after antigen stimulation [156]. In nasopharyngeal carcinoma, MIF promotes the formation and migration of Th17 cells mediated by the MIF-CXCR4 axis and dependent on the mTOR pathway [157].

Studies have shown that TA-MSCs, TA-MSCs-EVs and MIF affect the proportion balance of Th phenotype and promote the transformation of Th into promote tumor Th2 phenotype, however, the specific mechanism of action remains to be elucidated.

##### TA-MSCs, TA-MSCs-EVs and MIF promote immunosuppressive cells

Myeloid-derived suppressor cells (MDSCs). MDSCs, an immunosuppressive cell type in TME, inhibit anti-tumor immunity through a variety of mechanisms. Zhao et al*.* [158] confirmed that MSCs effectively facilitate accumulation of MDSCs to TME, as reported for TA-MSCs. CML-MSCs enhance immunosuppressive MDSCs production and aggregation, promoting tumor progression via upregulating immunomodulatory arginase-1 (Arg-1), IL-6, IL-1β, COX-2, and TNF-α in MDSCs and inhibiting anti-tumor immunity [40].

By carrying high levels of TGF-β, C1q, BC-MSCs-EVs not only enhance the immunosuppressive activity of MDSCs and induce conversion to type M2 macrophages expressing high levels of PD-L1, but also reduce PD-1 expression in infiltrating T cells through upregulation of TGF-β in MDSCs, thus inhibiting the anti-tumor immune response [122].

Huang et al. [159] demonstrated that MIF knockout inhibits not only recruitment of MDSCs but also tumor growth and metastasis, supporting the theory that MIF promotes tumor progression by exerting effects on MDSCs. MIF is considered to be a determinant of melanoma MDSCs differentiation and immune suppression [160]. MIF induces differentiation and stimulates chemotaxis of MDSCs through activating PI3K/AKT and p38/MAPK pathways in head-and-neck squamous cell carcinoma (HNSCC) [161]. MyD88-dependent MAPK and NF-κB pathways are also involved in MIF-CXCR2-mediated recruitment of MDSCs to bladder cancer TME [162]. A decrease in MIF in mouse BC tissue results in significant reduction of circulating MDSCs and its suppressive cytokines along with inhibition of growth and metastasis of BC in vivo, further indicating that MIF promotes tumor progression by regulating the number and function of MDSCs [129]. Interestingly, MIF secreted by CSCs is reported to enhance immunosuppression mediated by MDSCs through binding the CXCR2 receptor, which facilitates glioblastoma immune evasion [106]. A further study suggests that MIF relies on its tautomerase activity to promote myeloid cell differentiation into mononuclear MDSCs (mMDSCs), promoting the formation of immunosuppressed TME and consequently, tumor growth and metastasis [163].

The effects of TA-MSCs, TA-MSCs-EVs and MIF on MDSCs are reflected in promoting the aggregation of MDSCs in immunosuppressive TME and enhancing the function of MDSCs by upregulating the expression of immunoregulatory cytokines in MDSCs.

Regulatory T cells (Tregs). Tregs represent a special subgroup of T cells that play a pivotal tumor-promoting role. Tregs induce tumor progression by inhibiting anti-tumor immunity and are linked to poor prognosis [164]. MSCs have the capacity to generate Tregs [111, 150] and enhance their tumorigenic activity [110]. A recent study indicates that AML-MSCs and myelodysplastic syndrome (MDS)-MSCs efficiently induce Treg generation associated with sustained leukemic cell viability and proliferation [165]. BC-MSCs and GC-MSCs induce generation of Tregs and their production of IL-10, IL-17 and TGF-β, in turn, facilitating tumor cell progression [166, 167]. Mechanistically, IL-15 derived from GC-MSCs stimulates Tregs through activation of STAT5 in CD4^+^ T cells and upregulation of PD-1 [79].

Several reports have confirmed that MSCs-EVs enhance proliferation of Tregs and their immunosuppressive cytokines, including IL-10 [152, 168]. MSCs-EVs are reported to promote proliferation and immunosuppressive capacity of Tregs via upregulating IL-10 and TGF-β1 secreted from PBMCs [169]. Although the issue of whether TA-MSCs-EVs affect Tregs remains to be definitively established, findings to date clearly support the theory that TA-MSCs-EVs promote immunosuppressive TME formation by increasing the number and function of Tregs.

The use of exogenous MIF can increase the number of IL-10-producing Tregs via Toll-like receptor 2 [170] in the colon and peritoneal cavity of mice [171]. MIF promotes tumor growth by increasing Tregs production through modulation of IL-2 in a colon cancer model mouse model [172]. Previous studies have identified a role of MIF in recruitment of Tregs [173]. The use of MIF receptor antagonists led to a significant reduction in the number of Tregs in metastatic melanoma tissues in mice [146], indicating that MIF promotes tumor development via effects on Tregs.

Similar to the effect on MDSCs, TA-MSCs, TA-MSCs-EVs and MIF promote the production and recruitment of Tregs in immunosuppressive TME and enhance the expression of immunomodulatory factors, thus enhancing the tumor-promoting effect of Tregs.

Tumor-associated macrophages (TAMs). TAMs present in TME are influenced by various factors. These macrophages are usually induced and polarized to the tumor-promoting M2 phenotype. A number of studies indicate that TA-MSCs affect the quantity and function of TAMs. For example, GC-MSCs secrete IL-6 and IL-8 to activate the JAK2/STAT3 signaling pathway in TAMs and promote polarization to M2 phenotype, leading to enhanced proliferation and metastasis of GC cells [167]. In turn, EVs isolated from GC cells facilitate TAMs recruitment by activating NF-kB signaling in GC-MSCs while enhancing the phagocytic function of TAMs, upregulating IL-6 and IL-8 secretion, indicating the existence of a feedback loop between tumor cells and TA-MSCs [167]. Chemokines secreted by ovarian cancer (OC)-MSCs promote polarization of TAMs to M2 phenotype and chemotherapy resistance of OC cells [174]. Melanoma-MSCs have also been shown to induce TAMs polarization to M2 phenotype, stimulating angiogenesis and tumor progression [175].

Consistent with the above findings, MSCs-EVs have been shown to promote TAMs M2 polarization mediated by miR-21-5p [81]. Interestingly, MSCs-EVs could be efficiently internalized by TAMs, eliciting a switch from M1 to M2 phenotype [176]. In addition, BC-MSCs-EVs promote the polarization of M0 to M2 phenotype, upregulation of PD-L1 expression in M2 macrophages, and ultimately, growth and metastasis of BC [122].

Research on metastatic melanoma shows that MIF interacts with CD74 on TAMs, which triggers activation of AKT, ERK and downstream signal pathways and increases the expression of immunosuppressive factors in TAMs, including TGF-β, IL-10, IL-6, arginase-1 and PD-L1, enhancing their tumor-promoting effects [146]. MIF also triggers TAMs polarization into M2 tumor-promoting phenotypes via CD74 and CXCR7 signal transduction, which not only increases the pro-angiogenic potential of TAMs but also promotes MM cell survival, proliferation, tumor growth and metastasis [177]. Similar results have been obtained in lung cancer [178] and melanoma [179]. In addition, MIF promotes the recruitment and infiltration of macrophages in mice [151], potentially mediated by the MIF-dependent chemokines monocyte chemotactic protein-1 (MCP-1), CXCL10, and macrophage inflammatory protein 2 (MIP2) [180]. Conversely, a study on CLL mice reported that the absence or inhibition of MIF reduced the number and migratory activity of TAMs, leading to changes in their distribution, concomitant with increased apoptosis of CLL cells [181]. The promotor effect of macrophage recruitment is proposed to be related to the tautomerase activity of MIF [182].

These studies suggest that TA-MSCs and TA-MSCs-EVs promote the polarization of TAMs to the tumor-promoting M2 phenotype and increase the expression of immunosuppressive cytokines, MIF may play a role as a mediator in this process.

Neutrophils. Interactions of TA-MSCs with neutrophils are also reported to stimulate tumor progression. IL-6 secreted by GC-MSCs induces neutrophils chemotaxis by activating the STAT3-ERK1/2 signal cascade. Activated neutrophils promote expression of IL-8, TNF-α and CCL2, which protect against spontaneous apoptosis [183]. Furthermore, activated neutrophils induce MSCs to differentiate into CAFs, enhancing the growth and metastasis of GC [183]. Similarly, smoldering MM (SMM)-MSCs activate neutrophils and induce an immunosuppressive phenotype through TLR4 signaling. Activated neutrophils not only recruit immunosuppressive Tregs but also induce CD8^+^ T cells apoptosis via upregulation of reactive oxygen species (ROS), which promotes immunosuppressive TME formation and tumor progression [131]. Furthermore, BC-MSCs recruit CXCR2^+^ neutrophils into TME, inducing a significant increase in expression of metastasis-related genes (CXCR4, CXCR7, MMP12, MMP13, IL-6 and TGF-β) in tumor cells and consequent metastasis [184].

MSCs-EVs molecules have protective effects on neutrophils phagocytosis capacity and lifespan [185] as well as ROS production, along with inhibitory effects on neutrophils apoptosis [186].

The ability of MIF to promote neutrophils recruitment is documented in the literature [187]. MIF is highly expressed in head-and-neck cancer (HNC) and stimulates functions of neutrophils by enhancing their CXCR2-dependent recruitment and survival and release of CCL4 and MMP9, in turn, promoting tumor progression [188]. Neutrophils infiltration could also be induced by MIF produced by leukemia cells [189]. In an in vivo study, MIF-deficient mice showed markedly reduced neutrophils infiltration, tumor incidence and angiogenesis upon chronic UVB exposure [73]. Clinically, MIF and neutrophils counts are significantly positively correlated in various tumors, such as HCC [190] and GC [191], indicating that MIF in TME is potentially responsible for neutrophils recruitment. In addition, MIF has been shown to significantly inhibit neutrophils apoptosis via interacting with CXCR2 [192].

TA-MSCs, TA-MSCs-EVs and MIF mainly promote the recruitmention in TME, survival and secretory function of neutrophils, which not only inhibits the spontaneous apoptosis of neutrophils but also suppresses the killing of tumor cells by other anti-tumor immune cells.

Cancer-associated fibroblasts (CAFs). CAFs are involved in almost all stages of tumor progression and contribute significantly to tumor invasion, angiogenesis, regulation of TME metabolism, immune cell recruitment and reprogramming as well as chemotherapy resistance [193]. TA-MSCs can be induced to differentiate into CAFs by tumor cells or other components of TME to promote tumor progression. For example, in BC and CRC, MSCs are specifically recruited into TME where they are induced to differentiate into CAFs via paracrine pathways, such as TGF-β and PDGFR-β [194, 195]. Tumor-educated blood platelets (TEP) also induce MSCs differentiation into CAFs via stimulation of TGF-β expression [196]. In bladder cancer, TGF-β1 receptor and Smad2 are involved in differentiation of MSCs to CAFs, in turn, promoting tumor growth in vivo [197]. TGF has therefore been identified as the key signal in transformation of MSCs into CAFs. Lactate secreted by pancreatic cancer (PC) cells facilitates transformation of MSCs to CAFs by inducing an increase in 5-hydroxymethylcytosine (5hmC) levels [198]. Hepatoma-derived growth factor (HDGF) secreted by GC cells infected with *H. pylori* recruits MSCs and induces their differentiation into CAFs, leading to enhanced survival and invasive ability [199]. In addition, EVs derived from MM cells induce transformation of MSCs into CAFs with increased IL-6 secretion via delivery of miR-21 and miR-146a [200]. Chemotherapy agents, such as cytarabine and daunorubicin, are also reported to promote phenotypic transition from MSCs to CAFs [201].

##### TA-MSCs, TA-MSCs-EVs and MIF promote tumor development through regulation of other factors

Tumor angiogenesis. Tumor angiogenesis is a necessary condition for rapid tumor growth and metastasis. An earlier study utilizing high-resolution isoelectric focusing-coupled liquid chromatography tandem mass spectrometry (HiRIEF LC–MS/MS) achieved full characterization of 1927 proteins in MSCs-EVs, including several potential paracrine factors related to angiogenesis, such as PDGF, epidermal growth factor (EGF), FGF and NF-kB signaling pathways [202]. LC-MSCs promote tumor angiogenesis by secreting various pro-angiogenic factors (ASPN, Clusterin, vascular endothelial growth factor (VEGF), IL-8, Ang and PDGF-BB) [42]. Thyroid hormones additionally upregulate angiogenesis-related factors, such as Ang and insulin-like growth factor 1 (IGF1), and stimulate VEGF signaling in hepatocellular carcinoma (HCC)-MSCs via alpha(V)beta(3) integrin (avb3), which promotes tumor angiogenesis in vivo and in vitro [203]. Moreover, the pro-angiogenic effect of GC-MSCs is mediated by the NF-κB/VEGF pathway, which may also be involved in regulation of VEGF expression in tumor cells [204].

MSCs can additionally promote tumor vasculogenesis through generation of pericytes. For instance, PDGF-B derived from BC and PC cells promotes the transformation of MSCs into mature pericytes via interactions with neuropilin-1 (NRP-1) in MSCs [205]. Similarly, stromal cell-derived factor (SDF)-1α and PDGF-B released by tumor cells bind CXCR4 and PDGFR-β on MSCs, respectively, resulting in differentiation into pericytes, leading to vasculogenesis and tumor recurrence [206].

MSCs-EVs also transport angiogenic miRNA to vascular endothelial cells, which can promote angiogenesis [207]. Other studies have reported that MSCs-EVs increase VEGF expression in tumor cells by activating ERK1/2 [208] and NF-kB pathways [202], driving angiogenesis and tumor progression.

The serum MIF level in patients with esophageal squamous cell carcinoma (ESCC) is associated with that of VEGF and vascular density in tumor tissue [209]. MIF level in BC tissue is also positively correlated with IL-8 expression and tumor microvessel density (MVD) [34]. Furthermore, co-expression of MIF and its receptor CD74 in NSCLC is associated with greater tumor angiogenesis and angiogenic CXC chemokine levels [210]. Consistent with these findings, MIF is reported to promote tumor angiogenesis by upregulating VEGF in UVB-induced NMSC cells [73]. Additional studies showed that exogenous MIF induces BC cells to secrete VEGF and IL-8 [34]. Subsequently, similar results were reported in human rhabdomyosarcoma (RMS) [211] and intestinal tumor [212]. Importantly, inhibition of MIF expression in melanoma stroma decreases the response of tumor cells to hypoxia, suppressing VEGF expression and MVD in tumor tissue [213]. These results support the theory that MIF facilitates tumor growth by inducing angiogenesis.

Mechanistically, overexpression of MIF in NSCLC increases the phosphorylation level of JNK, c-Jun, and subsequent activity of the transcription factor AP-1 in a CD74-dependent manner, which promotes tumor angiogenesis by increasing expression of the angiogenic factors CXCL8 and VEGF [214]. MIF enhances tumor angiogenesis through activating the MAPK signal pathway by enhancing phosphorylation of p38-MAPK and p44/42 and expression of VEGF-C in BC cells [215]. MIF also promotes vasculogenic mimicry (VM) formation through the CXCR4-Akt-EMT pathway in glioblastoma to support malignant tumor progression [84].

The above studies indicate that TA-MSCs, TA-MSCs-EVs and MIF are correlated in promoting tumor angiogenesis. Both TA-MSCs and TA-MSCs-EVs can up-regulate the expression levels of angiogenic cytokines in tumor cells, MIF may play an important role in the process because it also upregulates angiogenic cytokines by binding to CD74 receptors and activating related signaling pathways.

PD-1 and PD-L1. PD-L1, one of the most important immune checkpoints, continuously inhibits the activation, proliferation and anti-tumor functions of T cells after binding PD-1, making it impossible to effectively recognize and kill tumor cells and resulting in tumor immune escape. Both PD-L1 and PD-1 are expressed in MSCs, although differences in expression levels in MSCs from different sources have been reported [111, 216]. TA-MSCs inhibit the killing effect of T cells on tumor cells via downregulating PD-1 and upregulating PD-L1 expression in TME, leading to tumor progression. PCa-MSCs significantly enhance PD-L1 expression under stimulation of IFN-γ and TNF-α, leading to inhibition of T cells proliferation and function [113]. Pro-inflammatory cytokines secreted by CD4^+^ T cells also upregulate PD-L1 in GC-MSCs through activation of the STAT3 pathway [217]. IL-8 secreted by GC-MSCs has been shown to upregulate PD-L1 in GC cells through the STAT3/mTOR-c-Myc axis, enhancing the cytotoxicity of CD8^+^ T cells against GC cells [218].

In addition, TGF-β and C1q contained in BC-MSCs-EVs induce upregulation of PD-L1 in MDSCs and M2 macrophages along with downregulation of PD-1 in infiltrating T cells, which maintains the immunosuppressive TME, thus overcoming the T cells-mediated anti-tumor immune response [122].

MIF interacts with its receptor CD74 to activate the IFNγ-JAK-STAT pathway, resulting in significant upregulation of PD-L1 in melanoma cells, which aids in tumor cell escape from the immune response and maintenance of immunosuppressive TME [219]. Data from a recent study indicate that MIF secreted by MM cells augments CD84 expression in TME, leading to upregulation of PD-L1 on MDSCs and suppression of T cells function, consequently promoting MM progression [220]. Neutralization of MIF is reported to inhibit PD-L1 expression in colon cancer-bearing mice, thereby suppressing tumor progression [128].

In terms of mechanism, TA-MSCs, TA-MSCs-EVs and MIF reduce the expression of PD-1 in immunosuppressive TME and PD-L1 in tumor cell and immunosuppressive cell, thereby inhibiting anti-tumor immunity and promoting tumor cell immune escape.

Fusion of MSCs and tumor cells. Multiple studies have shown that self-fusion and allogeneic fusion of tumor cells potentially give rise to the metastatic phenotype by generating widespread genetic and epigenetic diversity, which provides novel therapeutic opportunities [221]. Early reports have demonstrated the spontaneous formation of heterotypic hybrids between MSC and BC cells in vitro with predominantly mesenchymal morphological characteristics, mixed gene expression profiles and tumorigenicity in immunodeficient mice [222]. Although fusion of MSCs and BC cells is a rare event, hybrid cells exhibit stronger telomerase activity and proliferation ability, significant regulation of genes involved in EMT and increased expression of metastasis-associated S100A4 genes relative to parental cells. These results have been further confirmed in vivo [223]. Polyethylene glycol (PEG) stimulates fusion of human umbilical cord MSCs (UC-MSCs) and GC cells in vitro. The hybrid cells express high levels of stemness factors OCT4, Nanog, SOX2 and Lin28, resulting in enhanced migration, proliferation, and growth of gastric xenograft tumors in vivo [21]. Co-cultured MSCs and glioma stem cells can also fuse in vitro and these hybrid cells have been shown to promote tumor angiogenesis in vivo and ex vivo and tumorigenicity in vivo [224].

Malignant transformation of TA-MSCs. TA-MSCs promote tumorigenesis through spontaneous or malignant transformation induced by tumor cells or TME. Numerous studies have confirmed that after malignant transformation, TA-MSCs acquire tumor cell characteristics, such as increased proliferation, invasion/migration and pro-angiogenesis abilities, suppression of cellular senescence, and upregulation of protein and mRNA levels of tumor-related markers (glial fibrillary acidic portein (GFAP), CD133, Nestin and c-Myc) [225–227]. Malignant transformation of MSCs is affected by multiple factors, including microRNAs, oncogene expression, and methylation status [225, 228, 229].

In terms of the underlying mechanisms, Vishnubalaji et al*.* [230] showed that the Lin28b/let-7 axis is involved in the malignant transformation of hBM-MSCs. Akt, STAT3 and Wnt-β-catenin pathways are additionally associated with malignant transformation of MSCs [225, 226]. Moreover, low Rb and high c-Myc expression induce the expression of osteosarcoma-related markers, such as alkaline phosphatase (ALP), osteonectin and osteocalcin, which are related to malignant transformation of BM-MSCs into osteosarcoma (OS) cells [225]. The OS phenotype triggered by malignant transformation of hMSCs may be linked to overexpression of the activator protein-1 (AP-1) complex [231]. MSCs infected by Kaposi's sarcoma-associated herpesvirus (KSHV) could induce malignant transformation into Kaposi sarcoma cells by promoting mesenchymal-to-endothelial transition (MET) [232]. Malignant transformation of MSCs is also related to hypermethylated in cancer 1 (HIC1) and Ras-association domain family member 1A (RassF1A) methylation, which leads to decreased expression of tubulin [229].

## Potential relationships among TA-MSCs, TA-MSCs-EVs and MIF in tumors

As described above, TA-MSCs, TA-MSCs-EVs exert similar tumor-promoting effects via similar mechanisms as MIF. However, the potential associations between TA-MSCs, TA-MSCs-EVs and MIF are yet to be fully elucidated.

MIF mediates signal transduction mainly through CXCR2, CXCR4 and CD74 receptors. All three receptors are expressed on MSCs, indicating that MIF affects MSCs activity via interactions with different surface receptors [233–235]. MIFs secreted by AML cells promote IL-8 expression in TA-MSCs through CD74 receptors, supporting AML cell survival [236]. MIF-CXCR4 has been identified as the dominant chemotactic axis that drives MSCs homing to tumors. For example, MIF is reported to activate MAPK and its downstream signals via binding to CXCR4 on MSCs, inducing tumor homing of MSCs [237]. The other main effects of MIF on MSCs are anti-senescence and anti-apoptosis. MIF protects MSCs from spontaneous and hypoxia-induced senescence by reducing ROS production through regulation of AKT signaling [238, 239]. Separate experiments have shown that MIF reduces the effect of doxorubicin-induced cell senescence on MSCs by activating the PI3K/AKT pathway, which not only improves the proliferation, survival and activity of MSCs but also enhances the paracrine capacity of VEGF, bFGF, HGF and IGF, in addition to telomere length and telomerase activity [240]. Moreover, MIF activates the Wnt/β-catenin pathway by inhibiting lincRNA-p21, which suppresses the activities of caspase 3/7 and caspase 8 and reduces oxidative stress, thereby protecting BM-MSCs against hypoxia/serum deprivation-induced apoptosis [241].

MIF is expressed in MSCs from various tissue sources [242], including TA-MSCs [243]. Zhang et al. [244] demonstrated that MSCs suppress Dox-induced reactive oxidative stress and cardiomyocyte apoptosis by secreting high levels of MIF. MSCs cultured under hypoxia conditions upregulate MIF, which activates AKT signaling, leading to reduced mRNA expression of senescence-associated markers, maintenance of stemness and delayed senescence of MSCs [245]. However, no studies to date have explored the effects of MIF secreted by TA-MSCs on tumors. In terms of establishing whether MIF functions via EVs transport, Costa-Silva and colleagues reported that MIF is highly expressed in pancreatic ductal adenocarcinoma (PDAC)-derived EVs and its blockade prevents liver pre-metastatic niche formation and metastasis [246]. MIF is also expressed in EVs derived from MSCs and its EVs expression level is directly related to that in cells [247]. MIF affects the expression levels of genes and proteins in MSCs-EVs and inhibits apoptosis induced by oxidative stress through regulating the EVs-lncRNA-NEAT1/miR-142-3p/FOXO1 signaling pathway [248]. In turn, MSCs-EVs have been shown to affect MIF expression in cells and tissues, although the exact mechanisms remain unclear at present [249].

In a recent study, MIF was successfully upregulated in MSCs-EVs via plasmid transfection. MSCs-EVs with high MIF expression could significantly restore myocardial function in rats with myocardial infarction and inhibit mitochondrial division caused by hypoxia and serum deprivation by activating the AMPK signal pathway, leading to suppression of cardiomyocyte injury and apoptosis [247]. These findings suggest that TA-MSCs perform their tumor-promoting function through EVs MIF activity. However, consultation of abundant literature revealed no relevant research. Preliminary experiments by our group suggest that TA-MSCs secrete high levels of MIF in PC cells that play an important tumor-promoting role, which could be reversed by treatment with the EVs inhibitor GW4869 (unpublished data), confirming the above hypothesis.

## Discussion

In this review, we have comprehensively described the specific roles and mechanisms of action of MSCs, MSCs-EVs and MIF in TME. Notably, these molecules exert similar effects on tumor progression were similar, suggestive of potential interrelationships among MSCs, MSCs-EVs and MIF. In the previous table (Table 1), we summarized the effects of MSCs from different normal tissues on different types of tumors. It is not difficult to find that the effects of MSCs from different sources on tumors are different to varying degrees, which may be due to the fact that the protein and gene profiles of MSCs are partially changed by different cell microenvironments.

**Fig. 3:**
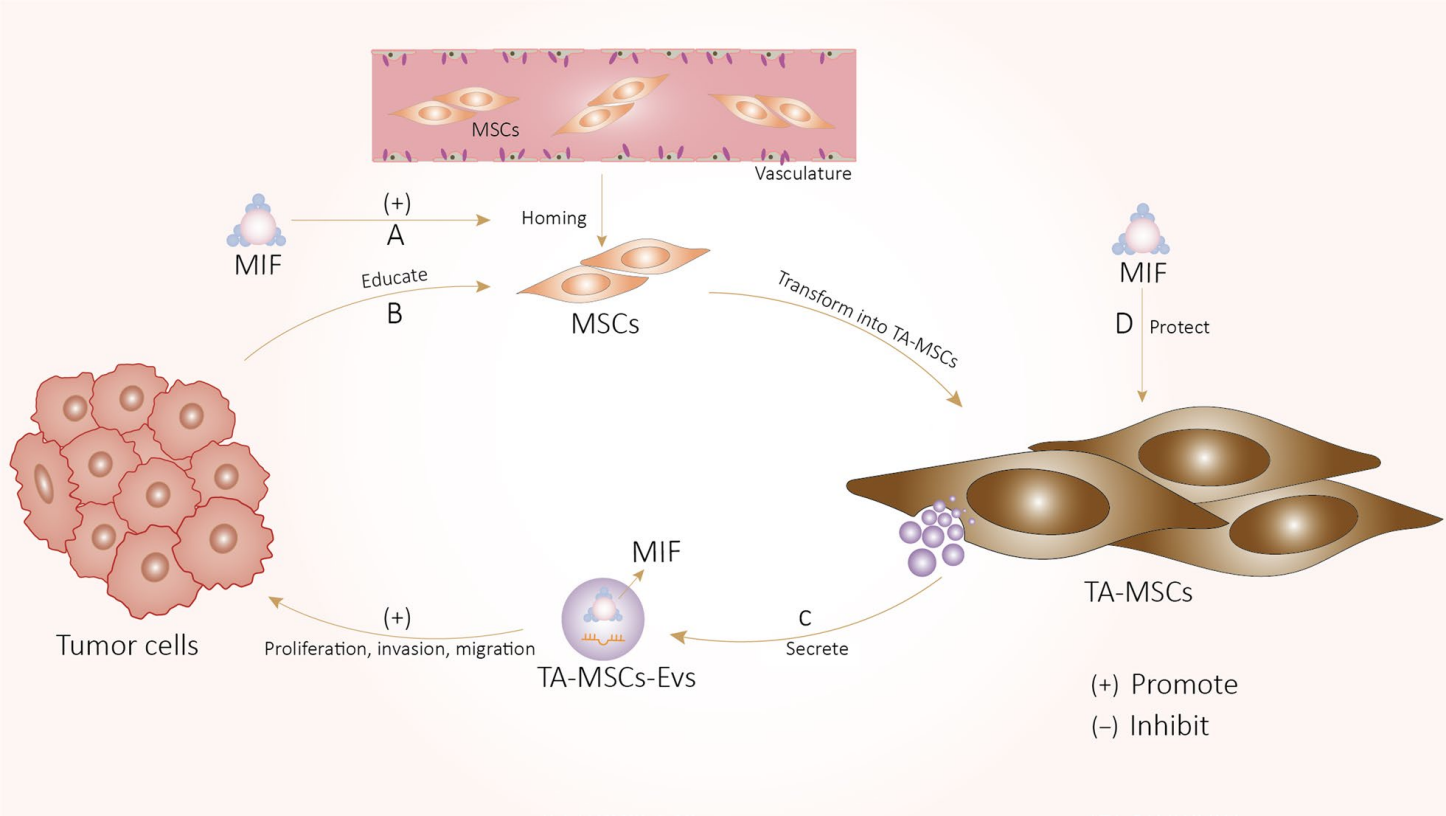
**A** Highly expressed MIF in tumors and stroma induce MSCs homing to TME. **B** After homing to tumor, MSCs are subsequently educated by tumor cells and TME to transform into TA-MSCs. **C** TA-MSCs-EVs promote the proliferation, migration and invasion of tumor cells through MIF transport, thus promoting tumor progression. **D** High levels of MIF in TME protect TA-MSCs from senescence and apoptosis

This review focuses on TA-MSCs derived directly from tumor tissue or induced differentiation by tumor microenvironment, based on the literature we have collected, almost all the relevant articles report that MSCs differentiate into TA-MSCs after being educated by tumor cells or TME, which drive tumor development. EVs clearly play a critical role in the tumor-promoting function of TA-MSCs. The experimental results we have conducted but have not yet published also confirmed that TA-MSCs have a significant promoting effect on pancreatic cancer, and this promoting effect is more obvious than BM-MSCs from normal tissues. Furthermore, the majority of studies support a tumorigenic role of MIF in TME. In view of the similarities in their effects and mechanisms of action, we speculate that TA-MSCs, TA-MSCs-EVs and MIF act cooperatively to promote tumor progression. We present a theoretical model based on the hypothesis that highly expressed MIF in tumors and stroma induce MSCs homing to tumors, which are subsequently educated by tumor cells and TME to differentiate into TA-MSCs. TA-MSCs transport MIF to tumor cells via EVs, facilitating tumor development. Our preliminary findings have partially confirmed this theory. During this process, high levels of MIF in TME not only induce MSCs to home to tumors and promote their transformation to TA-MSCs but also protect TA-MSCs from senescence and apoptosis and trigger their spontaneous malignant transformation. In addition, TA-MSCs secrete and transfer MIF to tumor cells via EVs, which directly activates the signaling pathways related to tumor progression. These proposed pathways may explain the comparable tumor-promoting functions of MSCs, MSCs-EVs, and MIF. Meanwhile, stress conditions (e.g., hypoxia) and cytokines (including tumor-derived MIF) in TME could induce TA-MSCs to secrete high levels of MIF, leading to upregulation of MIF in tumor cells that affects TA-MSCs function. Accordingly, TA-MSCs and tumor cells form a positive feedback loop of intercellular interactions through MIF that continuously promotes malignant progression (Fig. 3.). The potential associations among MSCs, MSCs-EVs and MIF provide promising therapeutic opportunities. As can be found from the previously summarized table (Table 1), MSCs that can inhibit tumor mainly come from human normal umbilical cord tissue, and existing studies have also found that UC-MSCs-EVs can inhibit metastasis of melanoma. Therefore, using UC-MSCs as a tool for tumor treatment is an idea supported by theoretical basis but yet to be further verified. The essence of EVs is a membranous vesicle-like structure, its formation and release are regulated by various enzymes. Some studies have found that GW4869 can inhibit the synthesis and release of EVs by cells, and GW4689 is a kind of neutral sphingomyelinase (N-SMase) inhibitor [250, 251]. Therefore, the development of an agonist that can enhance the activity of N-SMase may promote the synthesis and release of EVs. Drugs that cause MSCS to release large amounts of EVs and then collect them and inject them into the body may be used as a way to treat tumors. 4-IPP, a specific suicide substrate for MIF that binds covalently and irreversibly to MIF to inhibit its biologic activity [252]. This inhibitor is a small molecule with strong specificity that may be effectively loaded into MSCs-EVs by engineering techniques such as electroporation. Current studies have confirmed that MSCs have the homing property to tumor, MSCs-EVs may with similar tumor chemotaxis properties as MSCs, these EVs may home to the tumor site after entering the body. MSCs-EVs loaded with small-molecule inhibitors may be preferentially taken up by both TA-MSCs and tumor cells. Following ingestion, MIF small-molecule inhibitors suppress the functions of TA-MSCs and tumor cells by blocking MIF signaling and the positive feedback loop, thus inhibiting tumor progression.

## Conclusion

The activities of TA-MSCs, TA-MSCs-EVs and MIF in TME are potentially correlated. EVs, MIF axis and TA-MSCs form a positive feedback loop with tumor cells, which influences the occurrence and development of tumors. The functions of these three factors in TME may undergo dynamic changes with tumor growth and continuously affect tumor development.

## Data Availability

Not applicable.

## Abbreviations

AP-1: Activator protein-1
ATRT: Atypical teratoid/rhabdoid tumor
Bax: BCL2 associated X
BC-MSCs: Breast cancer-derived MSCs
BMP2: Bone morphogenetic protein 2
BTIC: Brain tumor-initiating cells
CAFs: Cancer-associated fibroblasts
CAFs: Carcinoma-associated fibroblasts
CCL5: C–C chemokine ligand 5
CDK: Cyclin-dependent kinase
CeCa-MSCs: Cervical cancer-derived MSCs
c-IAP2: Cellular inhibitors of apoptosis 2
CLL: Chronic lymphocytic leukemia
CML: Chronic myelomonocytic leukemia
COX-2: Cyclooxygenase-2
CRC: Colorectal cancer
CSCs: Cancer stem cells
CTL: Cytotoxic CD8 + T lymphocytes
CXCR2: C-X-C motif chemokine receptor 2
DCs: Dendritic cells
DCs: Dendritic cells
ECM: Extracellular matrix
ERK1/2: Extracellular signal-regulated kinase
EVs: Extracellular vesicles
FAK: Focal adhesion kinase
FGF: Fibroblast growth factor
FTO: Fat mass and obesity-associated protein
GADD45A: Growth Arrest and DNA Damage-Inducible Alpha
GA-MSCs: Glioma-associated mesenchymal stem cells
GBM: Glioblastoma
GC: Gastric cancer
hESC: Human embryonic stem cells
HCC: Hepatocellular carcinoma
HGF: Hepatocyte growth factor
HIC1: Hypermethylated in cancer 1
HIF: Hypoxia-inducible factor
HNC: Head-and-neck cancer
HNSCC: Head-and-neck squamous cell carcinoma
ICD: Immunogenic cell death
IFN-γ: Interferon-γ
IGF1: Insulin-like growth factor 1
IL-8: Interleukin-8
IR: Ionizing radiation
JAK2: Janus kinase 2
JNK: C-Jun N-terminal kinase
KS: Kaposi sarcoma
LPA: Lysophosphatidic acid
m6A: N6-methyladenosine
MAPK: Mitogen-activated protein kinase
MCP-1: Monocyte chemotactic protein-1
mDCs: Mature DCs
MDSCs: Myeloid-derived suppressor cells
METTL3: Methyltransferase-like 3
MIF: Macrophage migration inhibitory factor
MIP2: Macrophage inflammatory protein 2
miR: MicroRNAs
MM: Multiple myeloma
MMP-9: Matrix metalloproteinase-9
MSCs: Mesenchymal stem cells
MSCs-EVs: MSCs-derived EVs
mTOR: Mammalian target of rapamycin
NB: Neuroblastoma
NF-κB: Nuclear factor-κB
NK cells: Natural killer cells
NLT: Normal lung tissue
NMSC: Non-melanoma skin cancer
NPC: Nasopharyngeal carcinoma
NR3C2: Nuclear receptor 3C2
NR4A: Nuclear Receptor 4A
NSCLC: Non-small cell lung cancer
OC: Ovarian cancer
OCT4: Octamer-binding transcription factor 4
OS: Osteosarcoma
OSCC: Oral squamous cell carcinoma
PBMCs: Peripheral blood mononuclear cells
PC: Pancreatic cancer
PCa: Prostate cancer
PG: Prostaglandin
PGE2: Prostaglandin E2
PGK1: Phosphoglycerate kinase 1
PGK1: Phosphoglycerate kinase 1
PI3K/AKT: Phosphatidylinositol 3-kinase/protein kinase B
PKB/AKT: Protein kinase B
PLAGL1: PLAG1-Like Zinc Finger 1
PTEN: Phosphatase and tensin homolog
RassF1A: Ras-association domain family member 1A
ROS: Reactive oxygen species
rpS3: Ribosomal protein S3
SACC: Salivary adenoid cystic carcinoma
SCC-MSCs: Squamous cell lung carcinoma-derived MSCs
Smad5: Mothers against decapentaplegic homolog 5
SOX2: SRY-box transcription factor 2
STAT3: Signal transducer and activator of transcription 3
TAMs: Tumor-associated macrophages
TAMs: Tumor-associated macrophages
TA-MSCs: Tumor-associated MSCs
TGF-β1: Transforming growth factor β1
Th: Helper T cells
TME: Tumor microenvironment
TNF-α: Tumor necrosis factor α
Tregs: Regulatory T cells
UC-MSCs: Umbilical cord derived MSCs
uPA: Uridylyl phosphate adenosine
UVB: Ultraviolet B
VEGF: Vascular endothelial growth factor
ZEB1: Zinc finger E-box-binding protein 1
